# Intestinal intussusception related to colonic pedunculated lipoma: A case report and review of the literature

**DOI:** 10.1016/j.ijscr.2019.01.042

**Published:** 2019-02-10

**Authors:** Johanna Cordeiro, Leonardo Cordeiro, Paula Pôssa, Paula Candido, Alice Oliveira

**Affiliations:** a Departament of Radiology, Hospital Felicio Rocho, Av. do Contorno, 9530, Barro Preto, 30110-017, Belo Horizonte, MG, Brazil; b Departament of Surgery, Hospital Felicio Rocho, Av. do Contorno, 9530, Barro Preto, 30110-017, Belo Horizonte, MG, Brazil

**Keywords:** Intussusception, Lipoma, Colon, Abdominal pain, Multidetector computed tomography, Case report

## Abstract

**Introduction:**

Intestinal intussusception is a relatively common cause of bowel obstruction in children, however, it is a rare clinical entity in adults. When seen in adults, it is often caused by some underlying condition, usually of malignant origin. We present a case of intestinal intussusception caused by a benign and rare condition in the gastrointestinal tract.

**Case presentation:**

A 69-year-old male patient presented with diffuse abdominal pain for 2 months, which intensified in the last two days, associated with diarrhea, vomiting and weight loss, in addition to sporadic episodes of hematochezia. Colonoscopy revealed a vegetative-infiltrative lesion, occupying about 75% of the lumen of the colon, located in the hepatic angle, presumably neoplastic. A biopsy was performed, which revealed mild nonspecific chronic inflammation in activity, in fragments of colonic mucosa. CT scan demonstrated colo-colonic intussusception, associated to an oval formation presenting fat density, suggesting lipoma. The patient underwent laparotomy with intussusception reduction and right partial colectomy. The inspection of the specimen showed a yellowish, pedunculated lesion. Histopathology confirmed a 5.0 cm submucosal lipoma.

**Discussion:**

Intestinal intussusception is relatively frequent in children and is a rare clinical condition in adults at a ratio of 20:1.4. Sixty to sixty-five percent of the cases of intussusception in the large intestine have malignant etiology. Thus, lipoma as the main cause of colo-colonic intussusception in adults is an uncommon cause.

**Conclusion:**

Although rare, colonic lipoma should be considered as a differential diagnosis among the causes of large intestinal intussusception in adults.

## Highlights

• Intestinal intussusception is a rare clinical entity in adults.
• When seen in adults, intussusception is often caused by a malignant condition.
• Colonic lipoma is a rare benign condition that can cause intussusception in adults.
• Computed tomography is the most sensitive radiological modality for diagnosis.
• The treatment of intussusception in adults is almost always surgical.

## 1 Introduction

Intestinal intussusception is a relatively common cause of bowel obstruction in children; however, it is a rare clinical entity in adults, accounting for less than 1% of cases of bowel obstruction. When seen in adults, it is often caused by some underlying condition, usually of malignant origin. In up to 90% of cases, the causal factor can be demonstrated by imaging or surgical specimens [1].

Lipomas of the gastrointestinal tract (GIT) are rare benign tumors that may eventually serve as a point of intussusception [2]. They usually present as a sessile polypoid mass in the right colon and are rarely pedunculated [1,3].

This work has been reported in accordance with the SCARE criteria [4].

## 2 Case presentation

**Fig. 1:**
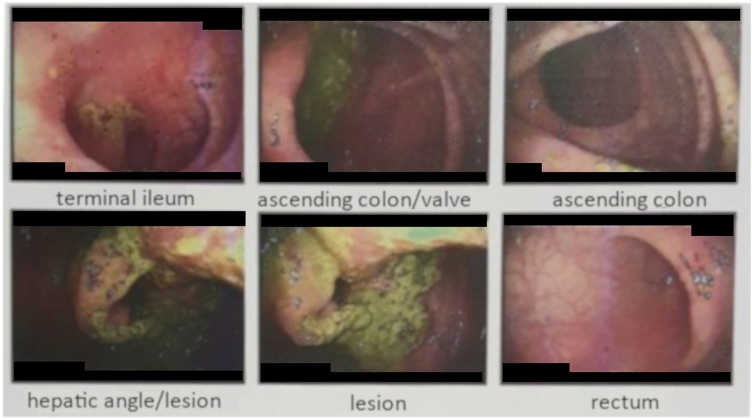
Vegetative-infiltrative neoplasic lesion with irregular contours, occupying about 75% of the lumen of the colon, located in hepatic angle.

**Fig. 2:**
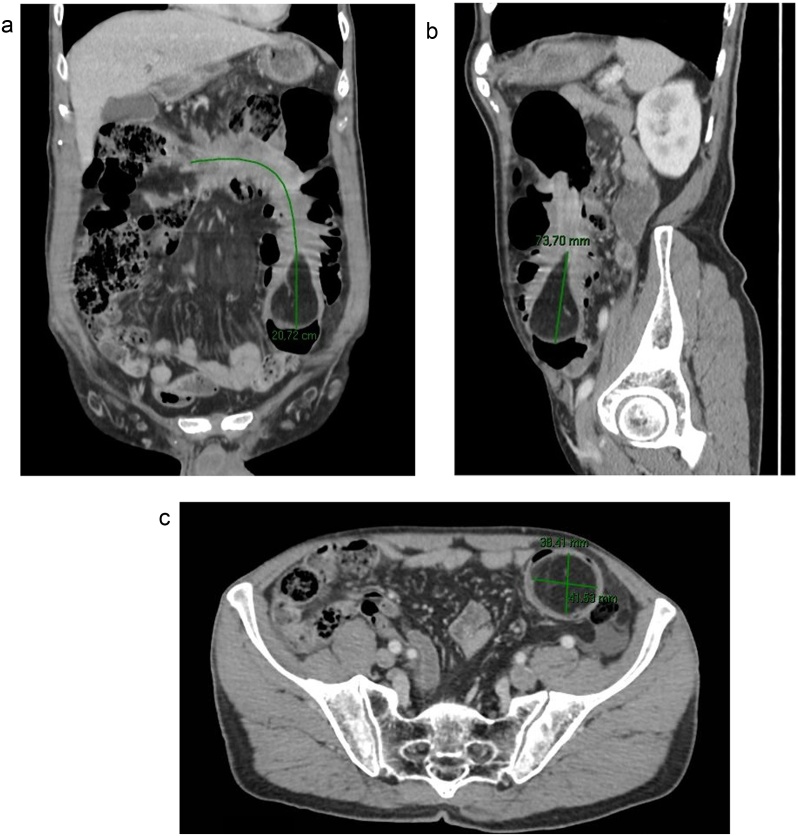
A – CT scan, coronal section in portal phase: Intestinal invagination extending longitudinally by 20.7 cm. The segments involved present normal parietal enhancement by the iodinated contrast media. B – CT scan, sagittal section in portal venous phase: Head of the intussusceptum related to the fat densit, round formation, measuring 7.3 × 3.8 × 4.1 cm. C – CT scan, axial section in portal venous phase: Head of the intussusceptum related to the fat densit, round formation, measuring 7.3 × 3.8 × 4.1 cm.

**Fig. 3:**
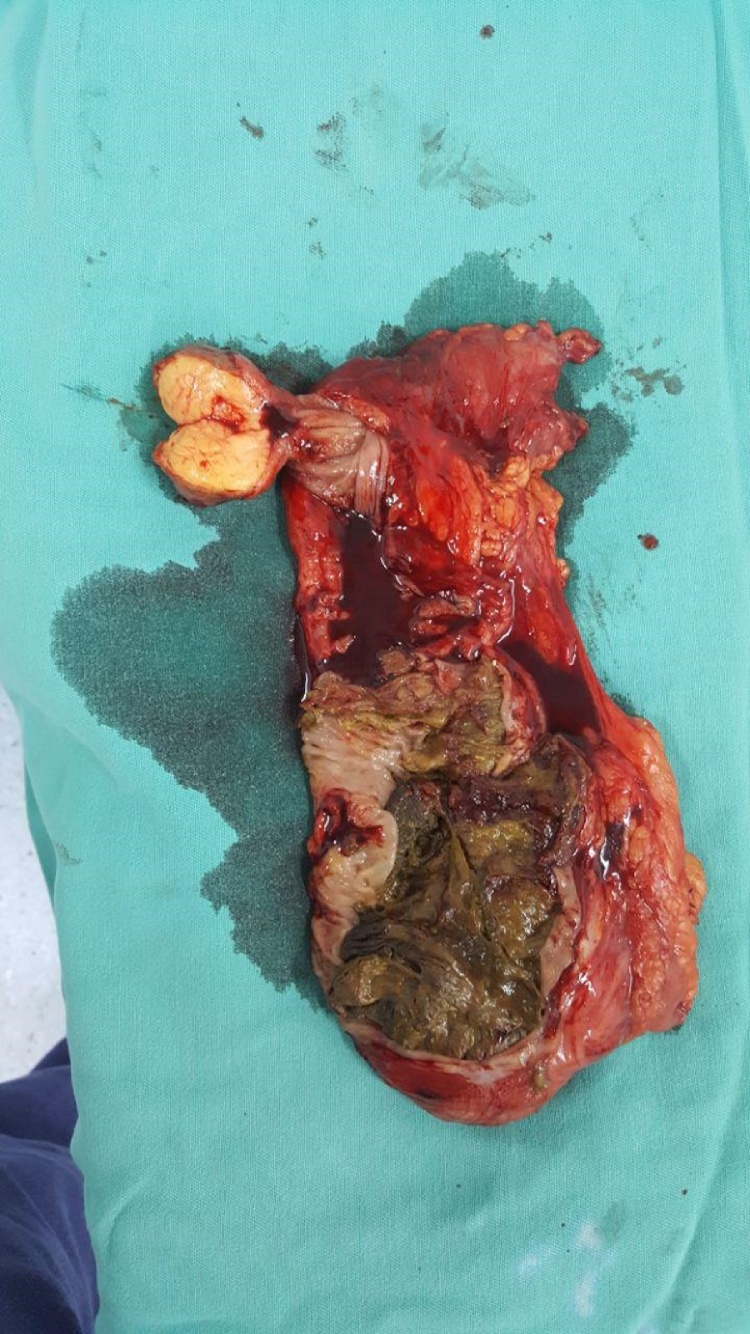
Surgical specimen demonstrating a resected segment of the colon and pedunculated lipoma.

A 69-years-old male patient presented to the hospital due to diffuse abdominal pain for 2 months, which intensified in the last two days, associated with diarrhea, vomiting and weight loss, in addition to sporadic episodes of hematochezia. The patient underwent colonoscopy (Fig. 1), which revealed a vegetative-infiltrative lesion, with irregular contours, hardened consistency, occupying about 75% of the lumen of the colon, located in the hepatic angle, presumably neoplastic. A biopsy was performed, which demonstrated mild nonspecific chronic inflammation in activity, in fragments of colonic mucosa. Computed tomography scan (CT) of the abdomen revealed colo-colonic intussusception, with the descending colon being the intussusceptive element, and the transverse colon being the intussusceptum with collapsed walls (Fig. 2A). At the distal end of the transverse colon there was an oval formation, presenting fat density, corresponding to the head of the intussusception and suggesting lipoma or some of its histological variants (Fig. 2B and C). The patient evolved to intermittent episodes of intestinal semi-obstruction. Although CT suggested that it was a lipoma, the macroscopic aspect of the lesion was suggestive of neoplastic lesion. This, together with the frequent episodes of intestinal semi-obstruction, led to a partial colectomy aiming patient safety. Surgery showed intussusception of the right/transverse colon, associated with a lesion located at the hepatic angle. Intussusception was reduced and a right partial colectomy was performed. The inspection of the specimen (Fig. 3) showed a yellowish, pedunculated lesion, measuring about 5.0 cm in diameter. Histopathology examination was compatible with colonic ulcerated submucous lipoma. The patient progressed favorably and had been discharged without complications.

## 3 Discussion

Intestinal intussusception is relatively frequent in children and is a rare clinical condition in adults at a ratio of 20:1.4. In adults it accounts for up to 5% of cases of bowel obstruction [5,6]. This is a rare condition in which a proximal segment of the intestine, called an intussusceptum, invades into an adjacent distal segment called intussusceptive [2,5,7]. The mechanism of intestinal invagination in adults is unknown in up to 20% of cases and is more likely to occur in the small intestine. On the other hand, it is believed that secondary intussusception starts from any pathological lesion of the intestinal wall that alters normal peristaltic activity and serves as a lead point, which is capable of initiating an invagination from one segment to another of the intestine [2,5].

The intussusception that occurs in the absence of a lead point is classified as primary or idiopathic, whereas in the secondary, a lead point is identified [6,8]. The primary usually occur in children and most commonly affect the small intestine [5]. In adults, the etiology can be identified in about 90% of cases, among them: organic lesions such as inflammatory bowel disease, bridles, Meckel’s diverticula, benign or malignant neoplastic lesions, or iatrogenic lesions [2,5,9,10].

Benign lesions are responsible for most cases of intussusception when it occurs in the small bowel, and only about 25–30% occur due to malignant lesions. On the other hand, 60–65% of cases of intussusception in the large intestine have malignant etiology [1,2]. Thus, colonic lipoma as the main cause of intussusception in adults is an uncommon cause [11].

Regarding the site of intussusception, there are four main types: (1) entero-enteric, involving only the small intestine; (2) colo-colonic, involving only the large intestine; (3) ileo-colic, involving the terminal ileum and ascending colon and (4) ileo-cecal, involving the ileocecal valve as the lead point. In adults, colo-colonic intussusception is the most common type [2,3,5].

GIT lipomas are benign tumors of mesenchymal origin [3]. They represent the most common cause of neoplasm of the GIT after adenoma [7]. Lipomas are more frequent in the large intestine, mainly in the right colon and in the cecum [3,7,12]. They are more frequent in women, with a peak of incidence between the 5th and 6th decades of life [7,12]. Usually, they present as a solitary, sessile or pedunculated polypoid mass emerging from the submucosa without any lesion of the mucosa, and may also affect the subserous layer and epiploic appendices [1].

Intussusception and intestinal obstruction caused by intraluminal lipomas are infrequent and their occurrence is directly related to their dimensions, usually when they present a diameter greater than 2 cm [7]. Those larger than 2 cm can cause intestinal obstruction without intussusception [10,13,14].

Lipomas of the colon are usually asymptomatic, especially smaller ones, being diagnosed incidentally during routine exams and rarely cause bleeding, obstruction and/or intestinal intussusception [2,7]. Intussusception is the most frequent complication of submucosal lipomas [3]. Only 25% of patients with colonic lipomas develop symptoms [2]. Lipomas greater than 4 cm are considered giants and more likely to develop symptoms, such as abdominal pain, changes in bowel habit, and weight loss. Less often, they may cause direct intestine obstruction or serve as a lead point for intussusception [12].

The early diagnosis of intussusception in adults is challenging because most cases present nonspecific signs and symptoms and have a chronic or subacute course [2]. The classic triad: (1) abdominal pain; (2) palpable abdominal mass; and (3) hematochezia, more frequently observed in children, is present in only 10% of the adults. In these, abdominal pain is the most common symptom, followed by nausea, vomiting and rectal bleeding [[1], [2], [3]].

Imaging methods can contribute greatly to the diagnosis of this condition. Abdominal CT scan is the most sensitive radiological modality [2,7], with sensitivity from 71.4% to 87.5%, and specificity, in adults, close to 100% [1]. The classic CT finding is the ‘target’ sign or ‘sausage-shaped’ soft tissue mass. The finding of mesenteric vessels around the lumen of the intestinal loop has also been described [2,3].

Ultrasonography (US) is another useful diagnostic method for both children and adults. It can show the classic ‘target’ sign in transverse sections and the ‘pseudo-kidney’ sign in longitudinal sections. US, however, has limitations inherent to the method, it’s highly operator-dependent and those classic signs are not always visualized [5].

Magnetic resonance imaging (MRI) is useful in the detection of benign fat lesions, such as intestinal lipomas, presenting a high signal in the T1-weighted sequences, similar to the sign of subcutaneous adipose tissue [1].

Colonoscopy can confirm the intussusception, indicate the lead point, and also represent a therapeutic option. However, it’s important to keep in mind that in some cases preoperative diagnosis can be difficult [1,5].

The treatment of intussusception in adults is almost always surgical, depending mainly on the size of the lipoma, location, preoperative diagnostic confirmation, or the presence of complications. Most authors recommend the surgical resection of lipomas greater than 2 cm, especially in older patients, in whom intussusception is more associated with malignancy [[1], [2], [3],^5^,^7^]. The prognosis of intussusception depends mainly on the causative factor of the lesion, and mortality from intussusception in adults increases from 8.7% in benign causes to 52.4% in malignant causes [2].

## 4 Conclusion

This case report demonstrates the possibility of intestinal intussusception being caused by intestinal lipoma, a condition of a generally benign nature. Preoperative diagnosis may be imaging or colonoscopy, and abdominal CT is the method of choice. Surgical resection is the best therapeutic option, especially in adults over 60 years old. Thus, colonic lipoma should be considered as a differential diagnosis among the causes of intestinal intussusception.

## Conflicts of interest

The authors declare that they have no competing interests.

## Sources of funding

This research did not receive any specific grant(s) from funding agencies in the public, commercial, or not-for-profit sectors.

## Ethical approval

We have approval from bioethical committee of Hospital Felicio Rocho, Belo Horizonte, Brazil.

## Consent

Written informed consent was obtained from the patient for publications of this case report and accompanying images. A copy of the written consent is available for review by the Editor-in-Chief of this journal on request.

## Author’s contribution

Johanna Alejandra V. Cordeiro: Wrote the paper.

Leonardo V. Cordeiro: Revised the manuscript.

Paula F. Pôssa: Collected the data for the case report.

Paula M. Cândido: Diagnosed the intussusception by CT scan.

Alice A. Oliveira: Operated on the patient.

## Registration of research studies

N/A.

## Guarantor

Johanna Alejandra V. Cordeiro.

## Provenance and peer review

Not commissioned, externally peer-reviewed.
